# The Qigong Wuqinxi for chronic obstructive pulmonary disease: Protocol for a systematic review and meta-analysis

**DOI:** 10.1097/MD.0000000000016633

**Published:** 2019-07-26

**Authors:** Feng Yu, Mengxue Xin, Nan Liu, Na Huang, Jianhui Lu

**Affiliations:** a The First Affiliated Hospital of GuangZhou University of Chinese Medicine; b Guangzhou University of Chinese Medicine, Guangzhou, China.

**Keywords:** chronic obstructive pulmonary disease, meta-analysis, protocol, systematic review, Wuqinxi

## Abstract

**Background:**

Chronic obstructive pulmonary disease (COPD) is a chronic and progressive disease that represents an important public health challenge nowadays. Despite the growing number of studies assessing the rehabilitation outcome of Wuqinxi for COPD, their many variables and observations are often explored with a relatively small sample size, accordingly maybe lead to potential false-positive results. The aim of this systematic review and meta-analysis is to evaluate the rehabilitation efficacy of Wuqinxi for COPD.

**Methods:**

A detailed search for articles up to June 2019 will be performed to identify randomized controlled trials for Wuqinxi in COPD. The following database will be used: PUBMED, Embase, Scopus, Web of Science, Google Scholar, Cochrane Library, Sino Med, Chinese National Knowledge Infrastructure, Chinese Science and Technology Periodicals Database, and Wanfang Database. Grey literature will be explored and the selection of studies, data extraction and validation will performed independently by 2 reviewers using predefined selection criteria and quality indicators. Stata V.13.0 and Review manager 5.3 software will be used for data synthesis, sensitivity analysis, subgroup analysis, and risk of bias assessment. We will use the grading of recommendations assessment, development, and evaluation system to assess the quality of evidence.

**Results:**

This research will update previous evidence summaries and provide a quantitative and standardized assessment of the rehabilitation efficacy of Wuqinxi for COPD.

**Conclusion:**

This systematic review will generate the latest evidence for determining whether Wuqinxi has a positive rehabilitation effect for COPD.

PROSPERO registration number: PROSPERO CRD 42019120960.

## 1 Introduction

Chronic obstructive pulmonary disease (COPD) is a chronic and progressive disease characterized by persistent respiratory symptoms and airflow limitation that is due to airway or alveolar abnormalities. From 1990 to 2015, the prevalence of COPD increased by 44.2%, it is the fourth leading cause of death nowadays, but it will be the 3rd by 2020.^[1,2]^ Therefore, COPD represents an important public health challenge. From a clinical perspective, COPD is usually divided into acute exacerbation episode and stable stage. the management strategy for stable stage is not limited to pharmacologic treatments and should be complemented by appropriate non-pharmacologic interventions.^[3]^ The benefits of pulmonary rehabilitation such as Self-Management and Integrative Care are significant,^[4]^ However, there are several potential barriers to the adoption of such measures, some of which relate to clinician attitudes towards its safety and efficacy. Previous study does not confirm that patients benefit from rehabilitation exercises and these rehabilitative means maybe difficult to generalize to real life.^[5]^ Health benefits from such rehabilitation project were counterbalanced by an unexpected and still unexplained higher mortality in patients with COPD participating in a comprehensive care plan.^[6]^ Wuqinxi (WQX) developed from the Chinese philosophy and Traditional Chinese Medicine theory. As one of the most widely practiced forms of traditional Chinese Qigong, it has been used to improve physical and psychological health for thousands of years.^[7]^ By mimicking the postures, movements, and bearing of the animals, along with their corresponding breath adjustment, practitioners will experience opening of the channels and network vessels, strengthening of the internal organs, and activation of the joints. Thus it is suitable for healthy people as well as patients with chronic diseases.^[8]^ A number of original studies were performed to examine the effects of WQX on the physical and psychological capacities for COPD patients,^[9–11]^ but their many variables and observations are often explored with a relatively small sample size that marks small effects as statistically significant, accordingly maybe lead to potential false-positive results. Although 1 relevant meta-analysis has been done, there are some drawbacks in the study, such as no eligibility criteria, only using a random-effects model rather than selecting models based on clinical heterogeneity, document quality was not evaluated by mainstream literature evaluation methods and the authors did not perform a comprehensive literature search.^[12]^ The absence of critically appraised evidence continues to exist, leaving little clarity for evidence-based clinical practice. A comprehensive, up-to date search examining WQX for the rehabilitation effect of COPD is justified.

## 2 Methods

### 2.1 Eligibility criteria

Eligible studies and trials will include published or unpublished reports in any language that assess the rehabilitation effect of WQX for COPD patients with the outcome of interest. The participants, interventions, comparisons, outcomes, and types of study criteria were agreed by the review researchers and provided in Table 1, as advocated by the 2009 Centre for Reviews and Dissemination handbook.^[13]^

**Table 1:**
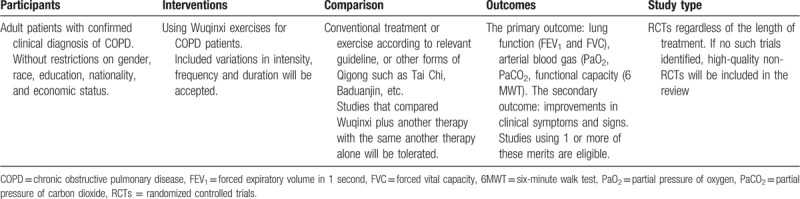
Participants, interventions, comparisons, outcomes, and study type of this systematic review.

### 2.2 Study type

This review is limited to randomized controlled trials (RCTs) firstly as these studies represent the highest level of evidence (level 1) currently available according to the Cochrane Handbook.^[14]^ Similarly, research methodologists consider the highest level of evidence of effectiveness is a well-performed systematic review that considers appropriate, high-quality RCTs. As scoping searches suggested a paucity of research in this area, we will apply a high level of sensitivity and low specificity to the search process and to include high-quality non-RCT studies, if no RCTs can be identified.

### 2.3 Search strategy and selection criteria

We will perform a comprehensive literature search from the following resources: electronic databases including PUBMED, Web of Science, Scopus, the Cochrane Library, google scholar, Embase, SinoMed, Chinese Science and Technology Periodicals Database, Chinese National Knowledge Infrastructure, and Wanfang Database for papers published from inception to June 2019. We will search manually for additional studies by cross-checking the reference lists of all included primary studies and lists of relevant systematic reviews. Grey literature sources including International clinical trials registry platform and 2 Qigong associations (China association of medical Qigong: http://www.cmqg.cn/, World Academic Society of Medical Qigong: http://www.wasmq88.com/sy) will be contacted for recent or unpublished papers. The search strategy will be developed by the research team in collaboration with an experienced librarian and checked by a referee according to the Peer Review of Electronic Search Strategy guidelines. The search items will include COPD, Chronic Obstructive Pulmonary Disease, COAD, chronic obstructive airway disease, Chronic Obstructive Lung Disease, chronic airflow obstruction, chronic airflow obstructions, pulmonary emphysema, Five animal show/exercise/play, Five poultry drills, Wuqinxi and Wuqinxi Qigong. Combined using Boolean logic (AND, OR, NOT). The following keywords Wuqinxi, Qigong, manxingzusexingfeibing, feiqizhong will be used for searching Chinese databases. To increase sensitivity, no restrictions were placed on language or study type. The exclusion criteria include full article not available or obvious methodological or statistical deficiencies. Literature will be searched by 2 authors (NH and MXX), and any inconsistencies are discussed with a third author (FY). Table 2 shows the search strategy for PPUBMED and web of science.

**Table 2:**
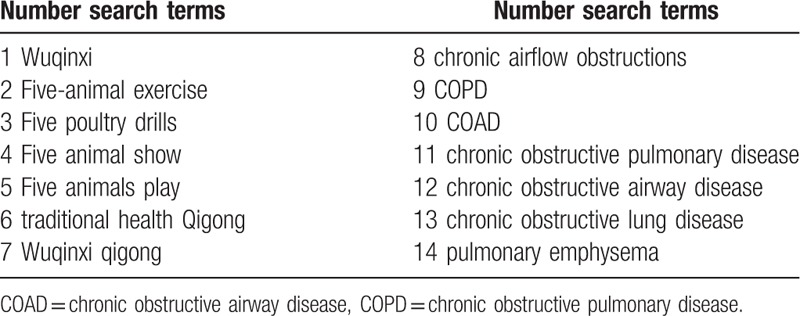
Search tactics for the PUBMED and web of science.

### 2.4 Study selection and data extraction

**Figure 1:**
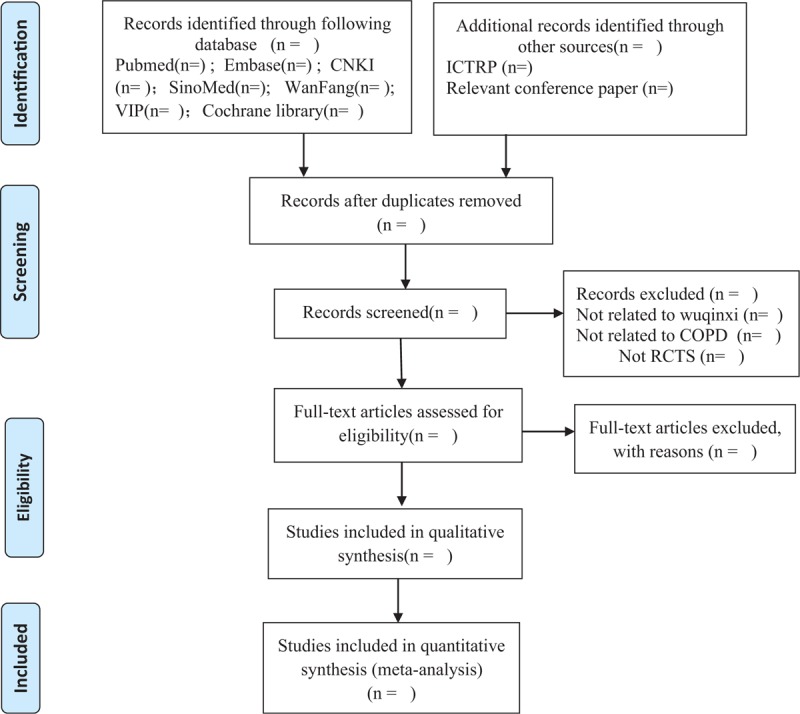
Preferred reporting items for systematic review and meta-analysis (PRISMA) flowchart.

Review process will be undertaken by 2 reviewers (HH and JHL) independently, applying blinding to reduce bias. Endnote V.X9 will be used to manage literature and remove duplications. The title and abstract of each article will be screened and assessed against predefined inclusion criteria. Full texts of all potentially relevant articles will then be assessed for inclusion. The reviewers will extract information about the study characteristics (design, sample size, duration of follow-up) participant demographics (age, sex), relevant medical conditions and outcome measures using a self-developed data extraction form. Discrepancies about inclusion in the meta-analysis will be discussed and settled by a third reviewer (NL). This protocol adheres to the preferred reporting items for systematic review and meta-analysis (PRISMA).^[15]^ The number of articles identified, screened, included and excluded, reasons for exclusion and to ascertain eligible studies will be described in a PRISMA flow chart (http://www.prisma-statement.org) (Fig. 1).

### 2.5 Risk of bias in included studies

Research may vary considerably in methodological, and flaws in the design or conduct of a study can cause bias, obscuring the benefit/harm of an intervention. The methodological quality of included RCTs will be assessed according to the Cochrane Handbook for Systematic Reviews of Interventions. Since it covers the assessment of several sources of bias, including random sequence generation, allocation concealment, blinding of outcome assessments, incomplete outcome data, and selective outcome reporting.

#### 2.5.1 Assessment of reporting biases

Begg rank correlation and Egger linear regression test will be used to assess funnel plot symmetry and interpret p values as showing statistical significance (*P* < .1).

#### 2.5.2 Addressing missing data or unclear measurement scales

We will try to contact corresponding authors (if email or telephone available) for missing data. If sufficient information cannot be obtained, we will analyze the available data. The potential influence of insufficient data on the review conclusions will be considered.

#### 2.5.3 Additional analyses

We will perform sensitivity analysis, meta-regression and subgroup analysis based on various study characteristics and sample size, such as study type, study quality, adjustment (or not) for confounders. A brief qualitative analysis of the evidence will be presented in narrative form if data extraction is insufficient or significant differences in study methods exist.

### 2.6 Data synthesis and analysis

We will evaluate clinical heterogeneity first. Clinical heterogeneity refers to the variation caused by different participants, interventions and different end-point indicators of the study. Statistical heterogeneity will then evaluated using the Cochran Q and I^2^ test if there is no clinical heterogeneity. For the *I*^2^ statistic, *I*^2^ thresholds of <25%, 25% to 49%, 50% to 75%, and >75% to represent low, moderate, high, and very high heterogeneity respectively. If statistical heterogeneity existed among studies, a random-effects model will be used to incorporate the heterogeneity while fixed effects model will be used if there is no heterogeneity. Partial pressure of oxygen and partial pressure of carbon dioxide are continuous outcomes, so the mean difference or the standardized mean difference are used for meta-analysis. All the results for risk ratios are displayed in the form of forest plots. If the test for heterogeneity is significant (*P* < .10), a brief qualitative analysis of evidence will be presented. We will analyze the data using Stata V.13.0 and Review Manage (v.5.3).

### 2.7 Quality of evidence

The grading of recommendations assessment, development, and evaluation (GRADE) offers a worldwide approach to guideline development for clinical practice, although it has the advantages and inherent limitations.^[16]^ The quality of the included evidence will be assessed by 2 reviewers independently according to GRADE approach. Reviewers will take into account limitations of the study, inconsistencies, indirect evidence, inaccuracies, and publication bias. Four levels of evidence quality (high, moderate, low, or very low) will be used.

### 2.8 Ethics and dissemination

This review will be published in a peer-reviewed journal and does not need ethical approval as there are no issues about participant privacy. Our findings will provide information about the rehabilitation efficacy of WQX for COPD patients.

## 3 Discussion

COPD patients may benefit from rehabilitation exercises, which represents integrated patient management that includes a range of healthcare professionals and sites, including hospital inpatient and outpatient settings.^[17,18]^ Currently, available rehabilitation treatment measures include education, self-management and integrative care all have limited efficacy, and prevention is paramount. Not all patients can benefit from these preventive approaches. WQX is an all-round exercise of human body that has been practiced for thousands of years. It was breed in the background of traditional Chinese philosophy and culture. The related theories of 5 elements and visceral manifestation also laid a solid foundation for its development. It includes bending forward, backward, and sideward, and twisting. So exerts a good stretching role for the chest, waist, leg muscle, ligaments waist, and legs. As a complementary, alternative medicine modality, WQX is practiced in many parts of the world for a variety of ailments, such as musculoskeletal disorders, psychosomatic diseases, insulin resistance, and respiratory diseases. Previous study has showed that WQX exercise could effectively improve the lung function and dyspnea symptoms of COPD patients in stable station, increase the movement ability and adjust patient's nourishment condition, therefore, prevent the decline of lung function, improve exercise ability and life quality, reduce death rate.^[19]^ Research has also indicated that WQX was helpful to improve the lung function and exercise tolerance of COPD patients during discharge transition period as it could promote their rehabilitation effects.^[9]^ However, the exact rehabilitation efficacy remains undefined. No reported trial has been large enough to accurately establish the efficacy of WQX for COPD with an acceptable degree of certainty. Before WQX can be recommended for routine use, we still require strong evidence. The aim of this review is to systematically assess all the currently available studies of WQX as a rehabilitation measurest for COPD, explore whether WQX exercise has a positive effect for COPD patients. The results of this review may help to establish a better approach to prevent the recurrence of the disease and suspend the deterioration of lung function and provide rigorous evidence for its clinical application and dissemination.

## Author contributions

**Conceptualization:** Jian Hui Lu.

**Data curation:** Meng Xue Xin.

**Formal analysis:** Feng Yu, Meng Xue Xin.

**Investigation:** Na Huang.

**Methodology:** Feng Yu, Na Huang.

**Software:** Jian Hui Lu.

**Supervision:** Nan Liu.

**Writing – original draft:** Feng Yu.

**Writing – review and editing:** Jian Hui Lu.
